# Atypical Presentation of Extensive Stanford Type A Aortic Dissection Initially Misattributed to Aspiration Pneumonia: A Case Report

**DOI:** 10.5811/cpcem.64043

**Published:** 2026-09-20

**Authors:** Margalit Goldgof, Ahmad Nama, Waseem Mujahed

**Affiliations:** * Hadassah University Medical Center Ein Kerem, Department of Emergency Medicine, Jerusalem, Israel; † Hebrew University of Jerusalem, Faculty of Medicine, Jerusalem, Israel

**Keywords:** aortic dissection, misdiagnosis, atypical presentation, aspiration pneumonia, case report

## Abstract

**Introduction:**

Acute aortic dissection is a life-threatening emergency that may present with nonspecific symptoms, particularly in older adults, leading clinicians to anchor on more common diagnoses.

**Case Report:**

An 85-year-old man presented with four days of cough, weakness, and constipation after a recent emergency department (ED) visit due to food impaction after he choked on a carrot. A chest radiograph obtained the day before admission showed left basal consolidation, prompting referral for suspected aspiration pneumonia. In the ED, he appeared comfortable with no complaints of chest pain. Physical examination noted mild tachycardia and wheezes bilaterally and treatment was initiated with antibiotics and bronchodilators. A low-voltage electrocardiogram led to immediate point-of-care echocardiography, which demonstrated a large circumferential pericardial effusion, a markedly dilated aortic root, and right ventricular collapse. Computed tomography angiography confirmed an extensive Stanford Type A aortic dissection.

**Conclusion:**

This case highlights how deceptively calm presentations can mask catastrophic disease and why clinicians must be ready to abandon their first diagnosis when the data demands it.

## INTRODUCTION

Acute aortic dissection carries high early mortality, particularly for Stanford Type A dissections, and outcomes depend critically on rapid recognition and surgical management. Classical symptoms include sudden, severe chest or back pain, pulse deficits, and hemodynamic instability, yet up to one-third of patients present atypically, especially older adults. Their complaints may be vague-weakness, dyspnea, abdominal discomfort, or gastrointestinal symptoms - leading to misdiagnosis such as pneumonia, acute coronary syndrome, or gastrointestinal disease.^1^,^2^

We report an extensive Stanford Type A aortic dissection initially attributed to aspiration pneumonia, emphasizing practical diagnostic lessons for emergency physicians: the need to maintain a broad differential; interpret electrocardiograms (ECG) meticulously (including low voltage), use bedside echocardiography early, avoid anchoring on an initial working diagnosis, and be aware of nuanced presentation in hemodynamic management when structural complications are present.

## CASE REPORT

An 85-year-old man with hypertension, hypothyroidism, and benign prostatic hyperplasia presented to the emergency department (ED) with four days of cough, generalized weakness, and constipation. He denied fever, chills, or dyspnea. Ten days earlier, he presented to another hospital with acute chest and epigastric discomfort immediately after swallowing a carrot, followed by two episodes of vomiting. At that visit, his blood pressure was 170/88 mm Hg; laboratory tests showed mildly elevated inflammatory markers, and a troponin level of 30 ng/L (upper limit of normal) (reference range, 0–35 ng/L). No imaging was performed, and he was discharged after symptomatic treatment.

The day before the current presentation, his family physician obtained a chest radiograph that showed left basal consolidation and blunting of the left costophrenic angle, raising concern for aspiration pneumonia and prompting referral to the ED. On arrival, the following vital signs were measured: heart rate 107 beats/minute (bpm); blood pressure 133/91 mm Hg; respiratory rate 18 breaths/minute; temperature 36.7 °C; and oxygen saturation 94% on room air. He appeared comfortable and cooperative. Cardiovascular examination revealed regular tachycardia and an apical heart murmur. Pulmonary auscultation demonstrated crackles at the right lung base and mild bilateral wheezes. The abdomen was soft, distended, and non-tender; there was no peripheral edema.

Laboratory studies showed no electrolytes abnormalities and stable creatinine 0.7 mg/dL (reference range, 0.55-1.02 mg/dL); liver enzymes were not elevated. Hemoglobin was 10.8 g/dL (down from 15 g/dL five months earlier) (12.4–16.1 g/dL), and platelets were mildly elevated at 478 ×10^9^/L (203–445^9^/L). Troponin was significantly elevated at 107 ng/L (55–58 ng/L). The initial ECG was of poor quality but showed no obvious ischemic ST-segment changes; however, repeat ECG was requested.

Given the presumed diagnosis of pneumonia, intravenous ceftriaxone and bronchodilator therapy were initiated. Because of persistent tachycardia and elevated troponin, computed tomography (CT) pulmonary angiography was ordered. Before imaging, a repeat ECG demonstrated markedly low voltage (Image 1), prompting point-of-care transthoracic echocardiography. The ECG revealed a large circumferential pericardial effusion with radiologic signs of tamponade (right ventricular collapse, video) and a markedly enlarged aortic root causing significant aortic insufficiency.

*CPC-EM Capsule*

What do we already know about this clinical entity?

*Type A aortic dissection often presents atypically in older adults and may mimic common conditions. Subtle symptoms do not exclude life-threatening pathology.*

What makes this presentation of disease reportable?

*The patient presented with classical symptoms of pneumonia: cough; weakness; and consolidation on chest radiograph; despite extensive Type A dissection with tamponade.*

What is the major learning point?

*Avoid anchoring on an initial diagnosis and be ready to pivot quickly when new data do not fit the presumed condition.*

How might this improve emergency medicine practice?

*Broad differentials, access to prior records, and point-of-care echocardiography improve diagnostic accuracy and can rapidly redirect care toward lifesaving intervention.*

Emergent CT angiography of the chest, abdomen, and pelvis demonstrated an extensive Stanford Type A aortic dissection originating at the aortic root and extending through the ascending aorta, arch, and descending thoracic aorta, and propagating into the left subclavian artery, celiac and mesenteric arteries, and left iliac artery (Image 2). Despite the extensive dissection, blood pressure remained stable and symmetric in both arms.

Cardiology and cardiothoracic surgery were urgently consulted. The patient underwent emergent surgical repair of the ascending aorta and aortic root. His postoperative course was uncomplicated, and he was discharged home 10 days later.

## DISCUSSION

Isolated case reports describe aortic dissection presenting with or mimicking pneumonia, aspiration events, or other pulmonary conditions, including dyspnea-dominant or painless presentations.^3^ Reports of dissection temporally associated with choking or food impaction are rare,^4^ but the mechanism is plausible: a sudden rise in blood pressure or intrathoracic pressure during a Valsalva-like event could precipitate dissection in a susceptible aorta. Extensive Type A dissections involving arch vessels, mesenteric arteries, and iliac arteries have been documented,^5^ however; they are uncommon. Survival to discharge, as in this case, is notable.

To understand how this diagnosis could have been recognized earlier, it is essential to break down the case and highlight the key clinical clues and lessons that emerged.

### Atypical Presentation and Symptom Interpretation

This case illustrates how aortic dissection can masquerade as common conditions in older adults. The patient’s presenting symptoms - cough, generalized weakness, and radiographic consolidation - were highly compatible with pneumonia, a far more frequent diagnosis in this age group. However, his earlier episode of abrupt chest and epigastric pain after swallowing food may have represented the onset of dissection rather than simple esophageal irritation.

Importantly, even his “minor” complaints, such as a week of constipation and abdominal distension, are retrospectively explainable by extension of the dissection into the mesenteric and celiac territories, with possible mesenteric hypoperfusion.^6^

### Troponin, Hemoglobin, and the First Presentation

At the initial hospital visit, the troponin level was at the upper limit of normal (30 ng/L) in a patient without known coronary disease. In isolation, a borderline troponin in an elderly hypertensive patient with chest/epigastric discomfort is an easy miss; many clinicians would reasonably attribute it to demand ischemia, gastroesophageal disease, or noncardiac causes. However, in retrospect, this value should not be dismissed outright—particularly when combined with severe acute symptoms and elevated blood pressure. Current guidelines emphasize that troponin elevation can occur in proximal aortic dissection and may be an indicator of dissection among patients with atypical symptoms.^7^

Similarly, the drop in hemoglobin from 15 g/dL to 10.8 g/dL over several months, although not dramatic, could be a subtle clue to chronic or subacute blood loss or ongoing pathology. That it was not recognized at the first encounter underscores how easily such trends are missed when prior records are not readily available or not reviewed under time pressure.

### Health-Record Fragmentation and Diagnostic Safety

The emergency team that ultimately diagnosed the dissection had access to prior hemoglobin values and the earlier troponin result; the first hospital may not have had the same longitudinal view or may not have had time to integrate it. Interoperable health records and concise, high-yield summaries of prior investigations are critical for pattern recognition and for avoiding missed opportunities in time sensitive conditions.

### Electrocardiogram, Low Voltage, and the Role of Point-of-Care Echocardiography

A central learning point from this case is the importance of meticulous ECG interpretation. Low-voltage ECG is often underappreciated but can signal pericardial effusion, obesity, chronic obstructive pulmonary disease, or infiltrative lung disease. In the context of unexplained tachycardia, elevated troponin, and a nonischemic ECG, low voltage should prompt urgent echocardiography to evaluate for pericardial effusion and structural pathology. In this patient, the decision to obtain point-of-care echocardiography based on low voltage was the pivotal step that redirected the diagnostic pathway and led to lifesaving intervention.

Point-of-care ultrasound has been shown to decrease misdiagnosis, as well as expedite diagnosis of ascending aortic dissection.^8^,^9^

### Cognitive Bias and the Need to Pivot from an Assumed Diagnosis

The case also illustrates diagnostic anchoring: once aspiration pneumonia was suspected - supported by cough, consolidation on chest radiograph, and wheezes - subsequent data were initially interpreted through that lens. Yet several features did not fit:

- Unexplained tachycardia in an afebrile, normoxic patient
- Elevated troponin without ischemic ECG changes
- Low-voltage ECG, suggestive of pericardial effusion
- Anemia and prior borderline troponin in a patient with recent chest/epigastric pain

Emergency clinicians must cultivate the habit of asking, “What doesn’t fit?” and be willing to rapidly pivot when new information conflicts with the working diagnosis. Literature on diagnostic error in emergency medicine repeatedly identifies anchoring and premature closure as major contributors to missed aortic dissection.^10^

### Nuanced Hemodynamic Management

Standard teaching emphasizes reducing aortic wall stress by lowering heart rate (< 60 bpm) and systolic blood pressure (< 120 mm Hg). Opioids can assist early by relieving pain and anxiety, thereby decreasing sympathetic drive. Vasodilators should only be introduced after adequate heartrate control (typically with beta-blockers) to prevent reflex tachycardia;^2^ however, in acute aortic regurgitation or tamponade, compensatory tachycardia is essential for maintaining cardiac output and suppressing it may precipitate rapid hemodynamic collapse.^10^ In complex presentations such as this, physiological priorities shift from limiting dissection progression to preserving the patient’s fragile compensatory state. Accordingly, the cardiothoracic surgeon recommended against hemodynamic manipulation. If hemodynamic collapse had occurred, controlled pericardial drainage (removing very small volumes of less than 30 mL) could have provided temporary stabilization, but definitive treatment remains expedited surgical intervention.^11^

## CONCLUSION

This case demonstrates how an extensive Stanford Type A aortic dissection can present with cough, generalized weakness, constipation, and radiographic consolidation - features easily attributed to pneumonia.

Key lessons include the following:

- All chest and epigastric pain and dyspnea should trigger consideration of aortic pathology, especially in older adults.
- Atypical symptoms in older adults - such as weakness, cough, or constipation—do not exclude life-threatening pathology, including aortic dissection.
- Low-voltage ECG should prompt urgent echocardiography, particularly in patients with chest or epigastric pain, dyspnea, or elevated troponin.
- Borderline troponin without ischemic ECG changes warrants a broader differential, including aortic dissection.
- Subtle symptoms such as constipation and abdominal distension may reflect mesenteric involvement in extensive dissections.
- Emergency clinicians must avoid anchoring on an initial diagnosis and be ready to pivot quickly when new data do not fit the presumed condition.
- Point-of-care echocardiography is a decisive tool that can rapidly redirect the diagnostic pathway and facilitate lifesaving intervention.

Early use of point-of-care echocardiography and a disciplined approach to conflicting clinical information can facilitate timely recognition and treatment of this life-threatening condition, as in this case, where the patient survived and was discharged home 10 days after surgery.

## Supplementary Information

Video. Point-of-care five-chamber echocardiographic view illustrating a large circumferential pericardial effusion (white asterisk). The right ventricle demonstrates diastolic collapse (arrow), and the aortic outflow tract is visualized anteriorly (black asterisk).

## Figures and Tables

**Image 1:**
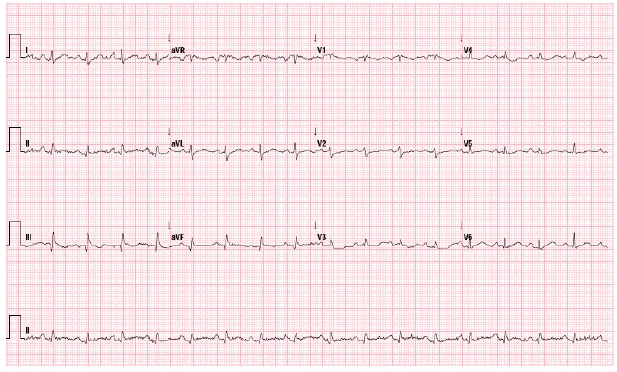
Twelve-lead electrocardiogram demonstrating sinus tachycardia with diffuse low-amplitude QRS complexes across limb and precordial leads. The overall low-voltage pattern is consistent with findings that may be seen in pericardial effusion or tamponade physiology.

**Image 2:**
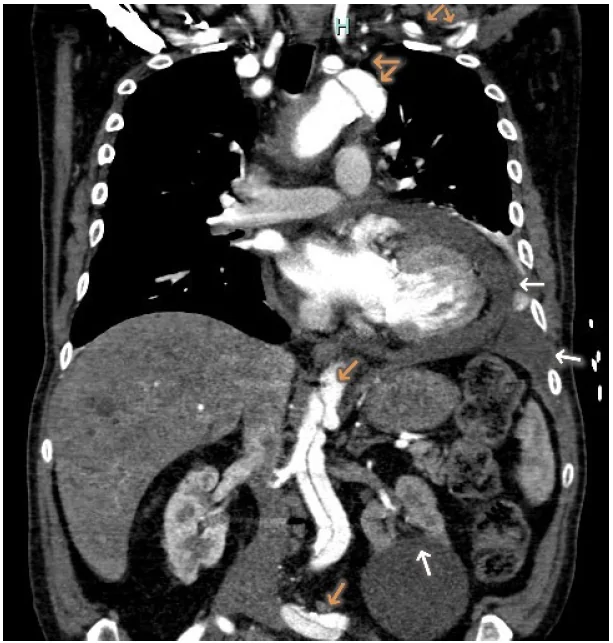
Computed tomography angiography image demonstrating an aortic root dissection extending to ascending aorta, left subclavian artery, descending aorta, and left iliac artery (yellow arrows). Circumferential pericardial effusion, left pleural effusion, and poor left renal perfusion were also noted (white arrows).
